# Diagnosis of Thyroid Nodules: Performance of a Deep Learning Convolutional Neural Network Model vs. Radiologists

**DOI:** 10.1038/s41598-019-54434-1

**Published:** 2019-11-28

**Authors:** Vivian Y. Park, Kyunghwa Han, Yeong Kyeong Seong, Moon Ho Park, Eun-Kyung Kim, Hee Jung Moon, Jung Hyun Yoon, Jin Young Kwak

**Affiliations:** 1 Department of Radiology, Severance Hospital, Research Institute of Radiological Science, Yonsei University College of Medicine, Seoul, Korea; 2 Health & Medical Equipment Business, Samsung Electronics Co., Ltd., Seoul, Korea

**Keywords:** Cancer imaging, Medical research

## Abstract

Computer-aided diagnosis (CAD) systems hold potential to improve the diagnostic accuracy of thyroid ultrasound (US). We aimed to develop a deep learning-based US CAD system (dCAD) for the diagnosis of thyroid nodules and compare its performance with those of a support vector machine (SVM)-based US CAD system (sCAD) and radiologists. dCAD was developed by using US images of 4919 thyroid nodules from three institutions. Its diagnostic performance was prospectively evaluated between June 2016 and February 2017 in 286 nodules, and was compared with those of sCAD and radiologists, using logistic regression with the generalized estimating equation. Subgroup analyses were performed according to experience level and separately for small thyroid nodules 1–2 cm. There was no difference in overall sensitivity, specificity, positive predictive value (PPV), negative predictive value and accuracy (all p > 0.05) between radiologists and dCAD. Radiologists and dCAD showed higher specificity, PPV, and accuracy than sCAD (all p < 0.001). In small nodules, experienced radiologists showed higher specificity, PPV and accuracy than sCAD (all p < 0.05). In conclusion, dCAD showed overall comparable diagnostic performance with radiologists and assessed thyroid nodules more effectively than sCAD, without loss of sensitivity.

## Introduction

Ultrasound (US) is the primary diagnostic tool for both the detection and characterization of thyroid nodules^1^. Several US features have been associated with thyroid cancer, including nodule hypoechogenicity, microcalcifications, irregular margins, and taller than wide shape^1,2^. However, interobserver variability is inevitable, with fair to moderate interobserver agreement being reported for most US features^3–5^. In addition, assessments based on individual US features have shown lower sensitivity and accuracy than assessments based on combined features, and therefore many professional societies and investigators have proposed US-based risk stratification systems that incorporate multiple US features for thyroid nodules^1,2,6–9^. Yet, such systems are also based on subjective assessments, and although reported values for interobserver agreement are somewhat higher, observer variation still exists for reporting US classifications and recommending biopsy^4,10–12^.

Computer-aided diagnosis (CAD) systems have been recently applied in the differential diagnosis of thyroid nodules, and hold potential to reduce operator dependence and improve the diagnostic accuracy of thyroid US. Previous studies have reported relatively high diagnostic performances of thyroid US CAD systems for thyroid malignancy, but were based on small study populations^13–17^. In addition, studies have compared the diagnostic performance of thyroid US CAD systems with those of one or two experienced radiologists, but their findings do not reflect actual clinical practice in which varying levels of experience are unavoidable^17–19^.

Several machine learning methods have been utilized for the development of thyroid US CAD systems, and the first commercialized thyroid US system using artificial intelligence utilized hand-crafted features and support vector machine (SVM) methods to classify thyroid nodules^20^. A prospective validation of this CAD system showed lower specificity (74.6%) and accuracy (81.4%) than those of an experienced radiologist, but similar sensitivity (90.7%)^19^. Recently, deep learning using convolutional neural networks (CNNs) has also been investigated as a tool for diagnosing thyroid nodules. Previous studies based on a retrospective collection of thyroid nodules reported sensitivities of 82.4–96.7% but still showed lower specificities (48.5–84.9%) than those of experienced radiologists^16,17^. With the recent rapid advances in machine learning technology and the inclusion of larger study populations, further improvement of such thyroid US CAD systems is expected^21^.

The purpose of this study was to develop a deep learning-based US CAD system for the diagnosis of thyroid nodules, and to prospectively compare its diagnostic performance with those of a SVM-based CAD system and radiologists.

## Results

The characteristics of the validation data set are described in Table 1. Among the 286 nodules, 130 (45.5%) were benign and 156 (54.5%) were malignant. Of the malignant nodules, 150 (96.2%, 150 of 156) were confirmed by surgical pathology and 6 (3.8%, 6 of 156) by malignant cytology. Of them, 149 (95.5%, 149 of 156) were papillary thyroid carcinoma, 6 (4.1%, 6 of 156) were minimally invasive follicular carcinoma, and one (0.6%, 1 of 156) was medullary carcinoma. For the 130 benign nodules, 58 (44.6%, 58 of 130) were confirmed as benign by surgical pathology, 66 (50.8%) by fine needle aspiration (FNA) cytology, two (1.5%) by core-needle biopsy (CNB) histology, and four (3.1%) by US findings of pure cystic nodules.

**Table 1:** Characteristics of the validation data set.

Characteristic	Experienced Radiologist	Inexperienced Radiologist	p-Value
Age (years)^a^	47.9 ± 13.3	45.9 ± 13.0	0.239
**Sex**^**b**^			
No. of men	38 (22.4)	14 (14.7)	0.134
No. of women	132 (77.6)	81 (85.3)	
Nodule size (mm)^c^	16.14 ± 0.80	16.49 ± 1.07	0.795
**Benign/Malignant**^**c**^			
No. of benign nodules	86 (46.7)	44 (43.1)	0.569
No. of malignant nodules	98 (53.3)	58 (56.9)	

^a^The independent two-sample t-test was used for comparison.

^b^The chi-square test was used for comparison.

^c^For nodule-based comparison, the generalized estimating equations (GEE) method was used.

### Overall diagnostic performance

Table 2 lists the performance measures of the deep learning-based US CAD system (dCAD), the SVM-based CAD system (sCAD), and all radiologists in diagnosing thyroid malignancy. The radiologists showed higher specificity (76.9% vs. 58.5%, p = 0.001), positive predictive value (PPV) (83.1% vs. 72.3%, p = 0.001) and accuracy (86.4% vs. 75.9%, p < 0.001) than sCAD. There was no significant difference in all performance measures between radiologists and dCAD (p value range, 0.137 to 0.862). dCAD also demonstrated higher specificity (80.0% vs. 58.5%, p < 0.001), PPV (84.5% vs. 72.3%, p < 0.001) and accuracy (86.0% vs. 75.9%, p < 0.001) than sCAD.

**Table 2:** Overall diagnostic performance of CAD systems and radiologists for diagnosing thyroid malignancy in the validation data set (n = 286).

Performance measures	Radiologists	dCAD	sCAD	p-Value	p-Value		
					Radiologists vs. dCAD	Radiologists vs. sCAD	dCAD vs. sCAD
Sensitivity	94.2% (89.3, 97.0)	91.0% (85.5, 94.6)	90.4% (84.7, 94.1)	0.137			
Specificity	76.9% (68.6, 83.6)	80.0% (72.4, 85.9)	58.5% (49.9, 66.6)	<0.001	0.431	0.001	<0.001
PPV	83.1% (76.5, 88.0)	84.5% (78.3, 89.2)	72.3% (65.6, 78.2)	<0.001	0.552	0.001	<0.001
NPV	91.7% (84.9, 95.7)	88.1% (81.0, 92.9)	83.5% (74.4, 89.8)	0.084			
Accuracy	86.4% (81.7, 90.0)	86.0% (81.6, 89.5)	75.9 (70.6, 80.5)	<0.001	0.862	<0.001	<0.001

Note – 95% confidence intervals are shown in parentheses.

### Diagnostic performance according to the experience level of the radiologists

Table 3 shows the performance measures of the CAD systems and radiologists according to different experience levels in thyroid imaging. The experienced radiologist group showed higher specificity (87.2% vs. 58.1%, p < 0.001), PPV (89.2% vs. 71.2%, p < 0.001), and accuracy (90.8% vs. 75.5%, p < 0.001) than sCAD. However, none of the performance measures significantly differed between the experienced radiologist group and dCAD. When comparing the two CAD systems, dCAD had higher specificity (84.9% vs. 58.1%, p < 0.001), PPV (87.3% vs. 71.2%, p < 0.001) and accuracy (88.0% vs. 75.5%, p < 0.001) than sCAD.

**Table 3:** Diagnostic performance according to the experience level of the radiologists.

Performance measures	Radiologists	dCAD	sCAD	*p*-Value	p*-*Value		
					Radiologists vs. dCAD	Radiologists vs. sCAD	dCAD vs. sCAD
**Nodules Assessed by Experienced Radiologists (n** = **184)**							
Sensitivity	92.9% (85.8, 96.6)	90.8% (83.3, 95.2)	90.8% (83.3, 95.2)	0.599			
Specificity	87.2% (78.3, 92.8)	84.9% (75.9, 90.9)	58.1% (47.7, 67.9)	<0.001	0.527	<0.001	<0.001
PPV	89.2% (81.5, 93.9)	87.3% (79.4, 92.4)	71.2% (62.7, 78.4)	<0.001	0.476	<0.001	<0.001
NPV	91.5% (83.1, 95.9)	89.0% (80.2, 94.2)	84.8% (73.2, 91.9)	0.318			
Accuracy	90.8% (83.3, 95.2)	88.0% (82.6, 92.0)	75.5% (68.8, 81.2)	<0.001	0.284	<0.001	<0.001
**Nodules Assessed by Inexperienced Radiologists (n** = **102)**							
Sensitivity	96.6% (87.5, 99.1)	91.4% (81.3, 96.3)	89.7% (78.9, 95.3)	0.145			
Specificity	56.8% (41.6, 70.9)	70.5% (55.9, 81.8)	59.1% (44.0, 72.7)	0.221			
PPV	74.7% (62.9, 83.7)	80.3% (68.8, 88.3)	74.3% (62.5, 83.3)	0.270			
NPV	92.6% (74.8, 98.1)	86.1% (70.7, 94.1)	81.3% (63.9, 91.4)	0.241			
Accuracy	79.4% (70.1, 86.4)	82.4% (73.9, 88.5)	76.5% (67.2, 83.8)	0.409			

Note – 95% confidence intervals are shown in parentheses.

In the inexperienced radiologist group, there were no significant differences in all of the performance measures between radiologists and each CAD systems (p value range, 0.145 to 0.409) (Table 3).

### Diagnostic performance in small thyroid nodules 1–2 cm in Size

Among the 286 thyroid nodules, 84 (29.4%) were 1–2 cm in maximum diameter. Of the 84 small thyroid nodules, 36 (42.9%) were benign and 48 (57.1%) were malignant. None of the patient and nodule characteristics of small thyroid nodules significantly differed between the experienced radiologist and inexperienced radiologist groups (Supplementary Table S1). For all small thyroid nodules (n = 84), there were no significant differences in diagnostic performance between radiologists and each CAD systems (Supplementary Table S2). However, when performing subgroup analyses according to the experience level of the radiologists, the experienced radiologist group demonstrated significantly higher specificity (95.2% vs. 61.9%, p = 0.011), PPV (95.2% vs. 71.4%, p = 0.023) and accuracy (95.4% vs. 76.7%, p = 0.006) than sCAD (Supplementary Table S3).

In the experienced radiologist group, radiologists tended to show higher specificity (95.2% vs. 81.0%, p = 0.089) and accuracy (95.4% vs. 88.4%, p = 0.084) than dCAD, although the differences were not statistically significant. In addition, dCAD tended to show higher specificity (81.0% vs. 61.9%, p = 0.095), PPV (84.0% vs. 71.4%, p = 0.089), and accuracy (88.4% vs. 76.7%, p = 0.056) than sCAD in the experienced radiologist group (Supplementary Table S3).

In the inexperienced radiologist group, there were no significant differences in all performance measures between radiologists and both CAD systems (p value range, 0.104 to 0.368).

### Incorrectly classified cases by radiologists or CAD systems

The radiologists incorrectly classified 39 cases (13.6%, 39 of 286) in the validation data set, of which there were 9 misclassified cancers. These consisted of four (44.4%, 4 of 9) cases of follicular variant of papillary thyroid carcinoma (FVPTC) and five (55.5%, 5 of 9) cases of minimally invasive follicular thyroid carcinoma.

sCAD incorrectly classified 69 cases (24.1%, 69 of 286) in the validation data set, of which there were 11 misclassified cancers. These consisted of eight (72.7%, 8 of 11) cases of conventional papillary thyroid carcinoma, one (9.0%, 1 of 11) case of FVPTC, one (9.0%, 1 of 11) case of minimally invasive follicular thyroid carcinoma and one (9.0%, 1 of 11) case of medullary thyroid carcinoma.

dCAD incorrectly classified 40 cases (14.0%, 40 of 286) in the validation data set, of which there were 12 misclassified cancers. These consisted of four (33.3%, 4 of 12) cases of conventional papillary thyroid carcinoma, two (16.7%, 2 of 12) cases of FVPTC, five (41.7%, 5 of 12) cases of minimally invasive follicular thyroid carcinoma and one (8.3%, 1 of 12) case of medullary thyroid carcinoma. Among the misclassified cancers by dCAD, 50% (6 of 12) were also misclassified by radiologists as well.

## Discussion

Our study results demonstrated that dCAD had performance comparable to radiologists for diagnosing thyroid malignancy, regardless of the experience level of the radiologists. Compared to sCAD, dCAD showed overall significantly improved specificity, PPV, and accuracy, while maintaining similar sensitivity. This indicates a clinically significant improvement in diagnostic performance, which supports the use of dCAD in clinical practice.

Several studies have investigated US CAD systems to diagnose thyroid malignancy^14,16,17,19,22^. SVM-based methods with textural features have been commonly used to classify thyroid nodules in these systems^14,19,22^, but have shown lower diagnostic performance than radiologists or have been based on studies retrospectively performed with a small number of thyroid nodules. Our study results were consistent with a prior prospective study evaluating the performance of sCAD, in which the CAD system showed similar sensitivity (90.7%) but lower specificity (74.6%) than an experienced radiologist^19^. Recently, Gao *et al*. assessed the diagnostic performance of an US CAD system based on a CNN framework, and reported similar sensitivity (96.7%) but lower specificity (48.5%) than an experienced radiologist^17^. Our method uses not only an input image, but also US feature information defined in TI-RADS, which radiologists have used to diagnose thyroid lesions in general. This approach may have contributed to the higher specificity in our study. Although the US features calculated by dCAD were not adjusted or used by the radiologist in this study, this approach may also potentially improve diagnostic performance in real clinical practice through interaction with users by providing TI-RADS US features as well as benign and malignant results. Nonetheless, in our study, dCAD still demonstrated higher specificity (80.0%) than the CAD system developed by Gao *et al*.^17^, while showing similar sensitivity, PPV, NPV, and accuracy. Our study is the first to report an US CAD system which shows comparable diagnostic performance with radiologists for diagnosing thyroid malignancy, and thus, has high potential for improving the diagnosis of thyroid nodules in actual clinical practice.

In this study, we further analyzed the diagnostic performances of radiologists and CAD systems according to experience level. Despite none of the patient and nodule characteristics differing according to the experience level of the radiologists, we found that only experienced radiologists exhibited higher specificity, PPV and accuracy than sCAD. In small thyroid nodules, experienced radiologists tended to show higher specificity and accuracy than dCAD, although without statistical significance. As the specificity range appeared to be lower in the inexperienced radiologist group in all nodules (56.8%) and the small thyroid nodule subgroup (53.3%), such differences may be attributed to the higher performance of experienced radiologists. Previous studies have shown that the accuracy of thyroid US depends on the experience of the interpreting physician^8^. In addition, whereas the CAD systems used two representative images of each thyroid nodule for assessment, the radiologists assessed thyroid nodules based on real-time US. Therefore, radiologists were able to make assessments based on more thorough imaging of each thyroid nodule, which may explain the higher performance seen in experienced radiologists. As the selection of representative images and semiautomatic segmentation would theoretically be influenced by the experience of the operator, this may also partially explain why there were no statistically significant differences between the performance measures of dCAD and sCAD in the inexperienced radiologist group. Another possible reason is the smaller sample size, as the number of included nodules in the inexperienced group was almost half the number included in the experienced group. Nevertheless, there was no significant difference in overall diagnostic performance between all radiologists and dCAD.

Interestingly, the specificity range of dCAD also appeared to be low in the inexperienced radiologist group in all nodules (70.5%) and the small thyroid nodule subgroup (46.7%). A possible reason for this is that the malignancy rate varies depending on the size of the lesion, and dCAD seems to automatically incorporate nodule size information from the input image itself to maximize overall performance, whereas sCAD is less affected by nodule size because the same feature values are extracted regardless of the size of the lesion. Therefore, the performance of dCAD may be more influenced by nodule size. On the other hand, experienced radiologists may select more representative images and achieve better lesion segmentation, which may lead to final assessments being less affected by size information.

Although most management guidelines recommend FNA for large thyroid nodules, controversy exists regarding the management of small thyroid nodules that are 1–2 cm in diameter^1,6,23–25^, which is why we chose to perform subgroup analysis for this size group. Yoon *et al*. reported that the criteria from Kim *et al*.^26^ showed the highest specificity (83.1%), PPV (59.6%), and accuracy (84.0%) among six previously published guidelines for thyroid nodules in this subgroup^27^, which was the same criterion used in our study. As the experienced radiologist group tended to show higher specificity and accuracy than dCAD in this subgroup, our results may suggest that the performance of the CAD system using CNN may be slightly lower in this size group – however, the number of small thyroid nodules 1–2 cm in diameter included in this study was small. Considering the ability of deep learning to discover intricate structure in large data sets^28^, future additional training with an even larger data set would likely further improve performance.

When reviewing the cases that were incorrectly classified by the radiologists or the CAD systems, we found that radiologists showed excellent performance in diagnosing conventional papillary thyroid carcinoma. This would be expected, as established suspicious US features are suggestive of this cancer type^2^. All of the misclassified cancers by radiologists were either FVPTC or minimally invasive follicular thyroid carcinoma, which tend to show more benign US features^29,30^. Although sCAD and dCAD showed a similar number of misclassified cancers, they differed in characteristics – conventional papillary thyroid carcinoma accounted for 72.7% (8 of 11) of the misclassified cancers by sCAD, whereas it accounted for only 33.3% (4 of 12) of the misclassified cancers by dCAD. Therefore, dCAD showed more similar classification results with radiologists, with 50% of its misclassified cancers overlapping with those of radiologists. Our results imply that deep learning-based methods not only improves diagnostic performance compared to SVM-based methods, but also in a direction similar to assessments made by radiologists.

Our study had several limitations. First, this study was conducted at a single academic center. As our institution is a referral center and we included thyroid nodules that underwent surgical excision or FNA, the overall malignancy rate was high (54.5%). In addition, a selection bias was inevitable since we excluded thyroid nodules with nondiagnostic or indeterminate cytology or histology. Further multi-center validation studies would be required to confirm our results. Second, the inexperienced radiologist group was composed of trainee fellows, who had varying experience with thyroid imaging during their residencies. Different results may be obtained in physicians with lower experience levels — however, our results still demonstrated differences in performances according to the experience level of the radiologists. Third, the experienced and inexperienced radiologist group assessed different thyroid nodules. Although characteristics of the validation data set did not differ between the two groups, this could have affected performance measures. Finally, a majority of the included malignancies were papillary thyroid carcinoma (95.5%), and thus, the diagnostic performance of the CAD systems may differ in populations with higher prevalence of other types of thyroid cancer.

In conclusion, the thyroid US CAD system using deep learning showed comparable performance with radiologists in diagnosing thyroid malignancy, regardless of the experience level of the radiologists. The newly developed CAD system is a promising tool for the assessment of thyroid nodules on US, by showing improved specificity, PPV and accuracy without loss of sensitivity.

## Methods

This prospective study was supported by a grant from Samsung Medison Co. in Seoul, South Korea, which also provided the equipment for this study. The study protocol was reviewed and approved by the Institutional Review Board of Severance hospital. Written informed consent was obtained from all patients before each US examination. All methods were performed in accordance with the relevant guidelines and regulations.

### Patients

**Figure 1:**
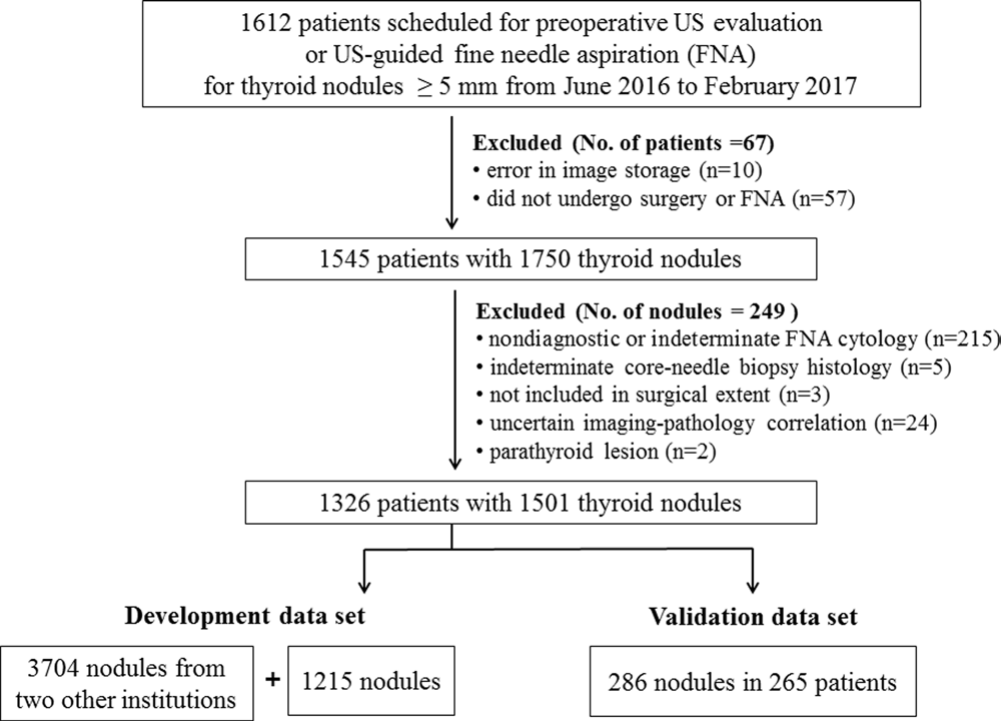
Flowchart of study population.

Patients were prospectively recruited at our hospital, a tertiary referral center, between June 2016 and February 2017. Potentially eligible patients were those requiring US for preoperative evaluation or those who underwent US-guided FNA for the diagnosis of thyroid nodules ≥ 5 mm. Patients with typical benign purely cystic nodules were also eligible. Only patients who received a malignant or benign diagnosis were included in the final study population. A malignant diagnosis was confirmed by surgical pathology or by CNB histology or FNA cytology. A benign diagnosis was confirmed by surgical pathology or CNB histology, FNA cytology, or US findings of benign purely cystic nodules^1^. In total, 1501 nodules in 1326 patients (mean age, 46.4 years ± 12.9; range, 19 to 85 years) with a definitive diagnosis were included (Fig. 1). All of the US images were acquired with a RS80A US system (Samsung Medison Co., Seoul, South Korea).

In this study, 1215 thyroid nodules diagnosed at our institution were used to develop a thyroid US CAD system using deep learning (S-Detect for thyroid, now loaded on RS85, Samsung Medison Co., Seoul, South Korea; referred to as dCAD), in addition to 3704 other thyroid nodules obtained from two other institutions using three US systems (iU22, Philips Healthcare, Bothell, WA, USA; EUB-7500, Hitachi Medical Systems, Tokyo, Japan; and RS80A, Samsung Medison Co., Seoul, South Korea). Therefore, US images of 4919 thyroid nodules from three institutions were used as a development data set for dCAD, which applied CNNs to classify thyroid nodules. An additional 286 thyroid nodules in 265 patients (213 women and 52 men; mean age, 47.2 years ± 13.2 [standard deviation]; mean nodule size, 16. 3 mm ± 10.9) diagnosed at our institution were used as an independent validation data set for which the performance of dCAD was prospectively evaluated.

### US examination and assessment by radiologists

Ten radiologists including four faculty members, with 5–20 years of experience in thyroid imaging, and six fellows training in thyroid radiology were involved in image acquisition. US examinations were performed with a 3–12-MHz linear high-frequency probe using a RS80A US system (Samsung Medison Co., Seoul, South Korea). US features of each thyroid nodule were prospectively recorded by the radiologist who performed the US or US-guided FNA, according to composition, echogenicity, margin, shape and calcifications^26^. Marked hypoechogenicity, microlobulated or irregular margins, microcalcifications, and nonparallel shape were considered as US features suspicious for malignancy^26^. When thyroid nodules exhibited at least one of the suspicious US features, they were assessed as “suspicious”. When thyroid nodules had no suspicious US features, there were assessed as “probably benign”. US-guided FNA was performed on nodules assessed as suspicious or on the largest nodule when there were only probably benign nodules.

### Data acquisition for the CAD system

**Figure 2:**
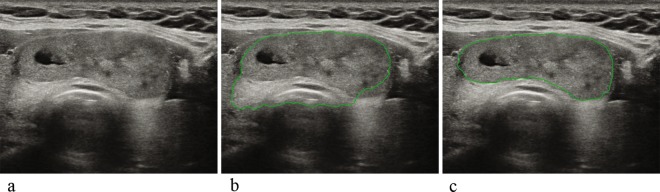
Example of ROI correction using semiautomatic segmentation by the first version of the CAD software (sCAD). (**a**) Image of a 51-year-old female patient with a 4.6-cm FNA-proven benign mass at the right thyroid. (**b**) When the user selected two points indicating the top-left and bottom-right points of a ROI box enclosing the thyroid nodule of interest, the initial semiautomatic segmentation results calculated by the CAD software included the adjacent normal thyroid tissue and trachea. (**c**) The user then manually selected a point at the correct nodule margin where the contour was miscalculated, and the CAD software correctly recalculated the contour of the nodule. The segmentation results shown in (**c**) were used for analysis. The nodule was assessed as possibly benign by both dCAD and sCAD.

The first version of the commercial thyroid US CAD software (S-Detect for Thyroid loaded on RS80A, Samsung Medison Co., Seoul, South Korea; referred to as sCAD) was integrated into the US system when US examinations were performed for this study. This CAD software let the user select two points indicating the top-left and bottom-right of a region of interest (ROI) box that included the thyroid nodule of interest in the US system^19^. Based on the ROI box, the CAD software calculated the nodule contour for segmentation. The software also provided a series of other candidates for nodule segmentation, from which the user was allowed to select if considered more accurate. When the semiautomatic segmentation included the adjacent normal thyroid tissue or neck structures, the user was allowed to manually select a point along the nodule margin, and the software would recalculate the nodule contour (Fig. 2). US features of the segmented nodule, including shape (ovoid-to-round or irregular), orientation (parallel or non-parallel), margin (ill-defined or microlobulated/spiculated or well-defined), echogenicity (hyper/isoechogenicity or hypoechogenicity), composition (cystic or partially cystic or solid) and spongiform appearance were quantified by the software. Consequently, the software automatically displayed the features of the nodule in real time, and presented a diagnosis as to whether the nodule was possibly benign or malignant. This process was performed twice for each nodule, on one representative image each for the transverse and longitudinal view, respectively. The US features and diagnosis provided by sCAD were later recorded for data analysis, but were not used by the radiologist for final clinical assessment.

### Development of thyroid US CAD system using deep learning (dCAD)

**Figure 3:**
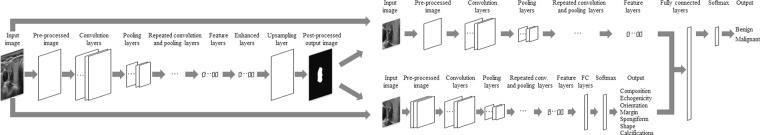
A conceptual figure of the development of the thyroid US CAD system using deep learning.

The development of dCAD consists of three steps. The first is the segmentation step to extract the boundaries of a lesion and the second is the classification step to extract the US features of the lesion. The last is the classification step to determine whether the lesion is benign or malignant (Fig. 3). The segmentation algorithm for extracting the boundaries of the lesion uses a modified algorithm based on a Fully Convolutional Network (FCN)^31^. The FCN is an algorithm for fully automated segmentation. However, lesions, especially thyroid lesions, are often not clear on US images, so a semi-automated segmentation method is used to reduce errors by specifying the location of the lesion with a bounding box. In the preprocessing module, the input image is transformed using the bounding box input selected by the user. That is, segmentation is performed on the area with some margins added to the bounding box. Because the lesion is located at the center of the modified image, the central region is enhanced in feature layers to improve segmentation performance.

In the second step, a new rectangular region is generated using the lesion boundary extracted in the first step, and this region is classified as an input image. In the pre-processing module, three images with different margins are generated for the input image. This is to analyze not only the lesion area but also the farther peripheral area together. We used AlexNet^32^, a type of CNN to output classification results for seven US features including composition (cystic or partially cystic or solid), echogenicity (hyper/isoechogenicity or hypoechogenicity) orientation (parallel or non-parallel), margin (ill-defined or microlobulated/spiculated or well-defined,), spongiform (appearance, non-appearance), shape (ovoid-to-round or irregular), and calcification (macrocalcifications, microcalcifications, no calcifications) for one image input.

In the third and last classification step, the lesion area which was obtained in the first step is used as an input image, and the US features which were obtained in the second step are integrated in the feature layer of the image to the lesion as benign or malignant. We modified GoogLeNet to take grayscale images as input and to have 2-class output of benign/malignant and removed two auxiliary classifiers^33^. We trained our network with ImageNet dataset which was converted into grayscale images and then used as a pre-trained model^34^. CNN training was implemented with the Caffe deep learning framework, using a NVidia K40 GPU on Ubuntu 12.04. A model snapshot with the lowest validation loss was taken for the final model. The learning hyperparameters were set as follows: momentum 0.9, weight decay 0.0002, and a poly learning policy with base learning rate of 0.25. The image batch size was 32, which was the maximum batch size that worked with our system.

### Evaluation of the thyroid US CAD system

Clinical validation of dCAD was performed with the independent validation data set. For each thyroid nodule, two representative images, one for the transverse view and one for the longitudinal view, were used for analysis. For each image, the developed dCAD presented a diagnosis as to whether the nodule was possibly benign or malignant. When at least one image was assessed as possibly malignant by dCAD, the nodule was classified as possibly malignant. The same approach was used when evaluating the performance of sCAD.

### Data and statistical analysis

We compared the diagnostic performance of dCAD in the validation data set, which utilized CNNs for the diagnosis of thyroid nodules, with those of sCAD and radiologists. Subgroup analyses were performed according to the experience level of the radiologists and nodule size, with an additional analysis performed for small thyroid nodules 1–2 cm in maximum diameter. The four faculty members (5–20 years of experience in thyroid imaging) were designated as experienced radiologists, and the six fellows training in thyroid radiology (1–2 years of experience in thyroid imaging) were designated as inexperienced radiologists for subgroup analyses. We compared demographics and nodule characteristics between the experienced and inexperienced radiologist group. For subject-based comparisons of demographics, the independent two-sample t-test and chi-square test were used. For nodule-based comparison of nodule characteristics, the generalized estimating equations (GEE) method was used.

The sensitivity, specificity, PPV, negative predictive value (NPV) and accuracy for thyroid malignancy diagnosis were calculated and compared by using logistic regression with GEE. Pairwise comparisons were performed for variables that showed statistically significant differences between the three groups (dCAD, sCAD, and radiologists). All statistical analyses were performed with SPSS software (version 23.0, IBM Corporation, Armonk, NY). A two-tailed *P* value of less than 0.05 was considered to indicate a statistically significant difference. In addition, we reviewed the cases that were incorrectly classified by the radiologists and both CAD systems.

## Supplementary information

Suppplemental tables

## Data Availability

The datasets generated during and/or analysed during the current study are available from the corresponding author on reasonable request.
