# Supplementary material for: Diagnosis of Thyroid Nodules: Performance of a Deep Learning Convolutional Neural Network Model vs. Radiologists
Source: Sci Rep. 2019 Nov 28;9:17843. doi: 10.1038/s41598-019-54434-1 (PMC6882804; doi:10.1038/s41598-019-54434-1)
Supplement: Supplementary file 1 — Suppplemental tables [file 41598_2019_54434_MOESM1_ESM.docx]

**Title: Diagnosis of Thyroid Nodules: Performance of a Deep Learning Convolutional Neural Network Model vs. Radiologists**

Contributing Authors:

Vivian Y. Park, M.D, PhD^1^, Kyunghwa Han, PhD^1^, Yeong Kyeong Seong, PhD^2^,

Moon Ho Park, PhD^2^, Eun-Kyung Kim MD, PhD^1^, Hee Jung Moon, MD, PhD^1^, Jung Hyun Yoon, MD, PhD^1^, Jin Young Kwak, M.D., PhD^1,*^

Affiliations:

^1^Department of Radiology, Severance Hospital, Research Institute of Radiological Science, Yonsei University College of Medicine,

^2^ Health & Medical Equipment Business, Samsung Electronics Co., Ltd.

**Table S1**. Characteristics of small thyroid nodules >10 mm and ≤ 20 mm in diameter

| **Characteristic** | **Experienced Radiologists** | **Inexperienced Radiologists** | **p-Value** |
| --- | --- | --- | --- |
| **Age (years)^a^** | 48.1 ± 13.3 | 47.9 ± 12.6 | 0.959 |
| **Sex^b^** |  |  |  |
| No. of men | 10 (75.6) | 5 (12.5) | 0.168 |
| No. of women | 31 (24.4) | 35 (87.5) |  |
| **Nodule size (mm)^c^** | 14.54 ± 0.46 | 14.39 ± 0.37 | 0.806 |
| **Benign/Malignant^c^** |  |  |  |
| No. of benign nodules | 21 (48.8) | 15 (36.6) | 0.257 |
| No. of malignant nodules | 22 (51.2) | 26 (63.4) |  |

^a^ The independent two-sample t-test was used for comparison

^b^ The chi-square test was used for comparison.

^c^ For nodule-based comparison, the generalized estimating equations (GEE) method was used.

**Table S2.** Diagnostic performance of CAD systems and radiologists for diagnosing thyroid malignancy in small thyroid nodules >10 mm and ≤ 20 mm in diameter (n=84)

| **Performance measures** | **Radiologists** | **dCAD** | **sCAD** | **p-Value** |
| --- | --- | --- | --- | --- |
| **Sensitivity** | 97.9%  (86.6, 99.7) | 93.8%  (82.3, 98.0) | 93.8%  (82.3, 98.0) | 0.263 |
| **Specificity** | 77.8%  (61.3, 88.5) | 66.7%  (50.5, 79.7) | 69.4%  (53.3, 81.9) | 0.352 |
| **PPV** | 85.5%  (73.5, 92.6) | 79.0%  (66.7, 87.6) | 80.4%  (68.1, 88.7) | 0.271 |
| **NPV** | 96.6%  (79.2, 99.5) | 88.9%  (70.7, 96.4) | 89.3%  (71.6, 96.5) | 0.200 |
| **Accuracy** | 89.3%  (80.6, 94.4) | 82.1%  (72.7, 88.8) | 83.3 %  (74.0, 89.8) | 0.141 |

Note – 95% confidence intervals are shown in parentheses.

**Table S3.** Diagnostic performance in small thyroid nodules >10 mm and ≤ 20 mm in diameter according to the experience level of the radiologists

| **Nodules Assessed by Experienced Radiologists (n=43)** | | | | | | | |
| --- | --- | --- | --- | --- | --- | --- | --- |
| **Performance measures** | **Radiologists** | **dCAD** | **sCAD** | **p-Value** | **p-Value^a^** | | |
|  |  |  |  |  | **Radiologists vs. dCAD** | **Radiologists vs. sCAD** | **dCAD vs.**  **sCAD** |
| Sensitivity | 95.5%  (73.9, 99.4) | 95.5%  (73.9, 99.4) | 90.9%  (70.0, 97.7) | 0.607 |  |  |  |
| Specificity | 95.2%  (72.8, 99.3) | 81.0%  (60.1, 92.3) | 61.9%  (41.3, 79.0) | 0.025 | 0.089 | 0.011 | 0.095 |
| PPV | 95.5%  (73.9, 99.4) | 84.0%  (65.3, 93.6) | 71.4%  (53.2, 84.6) | 0.009 | 0.109 | 0.023 | 0.089 |
| NPV | 95.2%  (72.8, 99.3) | 94.4%  (69.4, 99.2) | 86.7%  (59.5, 96.6) | 0.363 |  |  |  |
| Accuracy | 95.4%  (83.2, 98.8) | 88.4%  (75.7, 94.9) | 76.7%  (62.6, 86.7) | 0.015 | 0.084 | 0.006 | 0.056 |
| **Nodules Assessed by Inexperienced Radiologists (n=41)** | | | | | | | |
| **Performance measures** | **Radiologists** | **dCAD** | **sCAD** | **p-Value** | **p-Value^a^** | | |
|  |  |  |  |  | **Radiologists vs. dCAD** | **Radiologists vs. sCAD** | **dCAD vs.**  **sCAD** |
| Sensitivity | 100%  (100, 100) | 92.3%  (73.8, 98.1) | 96.2%  (77.2, 99.5) | 0.368^.^ |  |  |  |
| Specificity | 53.3%  (29.3, 75.9) | 46.7%  (24.1, 70.7) | 80.0%  (53.0, 93.4) | 0.141 |  |  |  |
| PPV | 78.8%  (61.6, 89.6) | 75.0%  (57.2, 87.1) | 89.3%  (71.5, 96.5) | 0.104 |  |  |  |
| NPV | 100%  (100,100) | 77.8%  (42.1, 94.4) | 92.3%  (60.9, 98.9) | 0.304 |  |  |  |
| Accuracy | 82.9%  (68.2, 91.7) | 75.6%  (60.2, 86.4) | 90.2%  (76.7, 96.3) | 0.105 |  |  |  |

Note – 95% confidence intervals are shown in parentheses.
